# Association between human papillomavirus and behaviour, clinicopathology, and cervical cancer outcome in Zimbabwean women: a cross-sectional study

**DOI:** 10.1186/s12978-025-02100-3

**Published:** 2025-10-07

**Authors:** Oppah Kuguyo, Alice Matimba, Nomsa Tsikai, Mugove Madziyire, Thulani Magwali, Collet Dandara

**Affiliations:** 1 Clinical Pharmacology Department, University of Zimbabwe Faculty of Medicine and Health Sciences, Mazowe Street, Harare, Zimbabwe; 2 SAMRC/UCT Platform for Pharmacogenomics Research and Translation, South African Medical Research Council, Cape Town, South Africa; 3 Radiotherapy Department, University of Zimbabwe Faculty of Medicine and Health Sciences, Mazowe Street, Harare, Zimbabwe; 4 Department of Obstetrics and Gynaecology, University of Zimbabwe Faculty of Medicine and Health Sciences, Mazowe Street, Harare, Zimbabwe; 5 Division of Human Genetics, Department of Pathology and Institute of Infectious Diseases and Molecular Medicine, University of Cape Town, Observatory, Cape Town, South Africa

**Keywords:** HPV, Africa, Cervical cancer, Sexually transmitted infections, Risk factors

## Abstract

**Background:**

Less than 10% of women infected with distinct human papillomavirus (HPV) develop cervical cancer, suggesting the need for secondary driving factors for carcinogenesis. This study describes factors associated with distinct HPV infections using cervical cancer cohort as a model. Moreover, we also determined the role of distinct HPV in the outcome of cervical cancer therapy.

**Methods:**

This cross-sectional study comprised of 240 Zimbabwean women aged > 18 years with histologically confirmed cervical cancer. Tumour tissue was obtained for genomic DNA analysis of 14 HPV genotypes. Demographic, behavioural and clinical information of study participants were collected for analysis. Logistic regression was used to determine factors associated with HR-HPV positivity, and outcomes of therapy.

**Results:**

The mean age(SD) of the group was 52(12) years. High HIV-positivity (48%) and sexually transmitted infection history (30%) were observed. HPV16 (35%), HPV35 (33%) and HPV18 (32%) were most prevalent. In unadjusted analyses, STI history (OR = 2.5, 95% CI 1.8–4.4, *p* < 0.01) was associated with HPV51 infections. Alcohol consumption was associated with HPV35 (OR = 1.93, 95% CI 1.1–4.9, *p* = 0.049) and HPV58 (OR = 2.5, 95% CI 1.6–3.8, *p* = 0.030). Smoking history was associated with HPV39 (OR = 5.8, 95% CI 2.0–7.8, *p* = 0.001) and HPV56 (OR = 2.0, 95% CI 1.2–4.3 *p* = 0.001). In adjusted analyses, HPV35 positivity was associated with high BMI (aOR = 1.4; 95% CI 1.1–1.7, *p* = 0.010). No HPV was associated with outcome.

**Conclusions:**

We describe the association between high BMI and smoking with distinct HPV genotypes. There is need for further research in a larger cohort to build predictive algorithms towards strengthening existing preventive, screening and predictive outcome interventions for HPV.

**Supplementary Information:**

The online version contains supplementary material available at 10.1186/s12978-025-02100-3.

## Background

Cervical cancer continues to pose a public health threat to women across the globe. Cervical cancer is the 4th most common cancer detected among women globally, with an estimated 604,000 new cases and 342,000 deaths per annum [1]. The highest cervical cancer burden and mortality rates in the world are in Africa, due to low implementation of prevention, screening and early detection programs coupled with the synergistic HIV epidemic [2]. Zimbabwe records the fourth-highest global cervical cancer incidence rates and mortality rates that are nearly seven times the global average [3, 4]. Cervical cancer is the most common cancer in Zimbabwe, with an estimated 3043 new cases and 1976 deaths per year [4]. Low implementation and uptake of screening and prevention interventions are reported in Zimbabwe, due to wide-ranging factors, including socioeconomic and health-system-related barriers, leaving many women susceptible in Zimbabwe [5, 6].

Infection with human papillomavirus (HPV), plays a critical role in cervical cancer aetiology [7]. HPV infections are ubiquitous and sexually transmissible, affecting more than half of all sexually active individuals in their lifetime. HPV infections normally resolve naturally within 24 months; however, highly oncogenic HPV genotypes can persist to precancerous lesions or cancer [7, 8]. There are 15 highly oncogenic HPVs, also termed high-risk HPVs (HR-HPVs), namely HPVs 16, 18, 31, 33, 35, 45, 51, 52, 56, 58, 59, 66, 68, 73 and 82. Only a small proportion of HR-HPV infections develop into precancerous lesions (10%) or cancer (< 1%), highlighting the possible role of secondary driving factors in persistence [9, 10].

Genetics, age, smoking, parity, use of oral contraceptives, diet, co-infection with HIV or other sexually transmitted infections (STI), sexual behaviour, having multiple sexual partners, and uncircumcised sexual partners are some factors that have been shown to drive the persistence of HR-HPVs [11, 12]. Emerging evidence shows geographical and sociocultural diversity in the prominence of distinct secondary driving factors for HPV persistence. For instance, in Asian populations, smoking was associated with HPV persistence and high oncogenic viral load, while in other populations, no relationship was found [13–16]. Among African populations, early sexual debut, early marriage and polygamy have been reported as predominant HPV propagators, and contrastingly, in Indian populations, multiparity was reported to be a more prominent propagator for HPV [17–23].

Moreover, distinctive HR-HPV genotypes have been shown to influence tumour histology and prognoses of cervical cancer, however, conflicting results have been obtained in diverse population groups [23–25]. HPVs 16, 18, 31, 45 and 52 have been associated with poor prognosis, while other studies correlate HPV58 with improved response to treatment in cervical cancer [26–32]. This data highlights the importance of analysing the role of HPV genotypes as potential prognostic biomarkers in different populations.

Environmental and sociocultural factors have been shown to contribute to the vaginal microbiota, consequently, resulting in diverse HPV profiles observed between populations. However, not many studies describe drivers for HPV persistence in African populations. The primary objective of the current study is to describe the relationship between clinical, pathology, and behavioural factors with distinctive HR-HPV infections. The secondary objective of the study is to determine the role of distinct HR-HPV in predicting the outcome of therapy among cervical cancer patients. These data can be applied to identify modifiable factors associated with HR-HPV infections, towards developing interventions that can augment the available screening and preventive measures.

## Methods

### Ethics

All study protocols and procedures were following the Helsinki Declaration of 2013. Ethical approvals for this study were obtained from the institutional review board, Joint Research Ethics Committee for the University of Zimbabwe Faculty of Medicine and Health Sciences and Parirenyatwa Group of Hospitals (JREC/412/16), as well as the national review board, Medical Research Council of Zimbabwe (MRCZ/A/2153). A materials transfer agreement for the study was obtained from the Research Council of Zimbabwe (permit number: 03351).

### Study setting

Participants were recruited from the Parirenyatwa Group of Hospitals Radiotherapy and Chemotherapy Centre (RTC), a public outpatient oncology facility in Harare, Zimbabwe. Participant information was collected from the RTC patient records during recruitment. Tumour biospecimens were collected from participating pathology laboratories, namely, Lancet Pathology and the Parirenyatwa Group of Hospitals Department of Pathology. Biospecimens were transported to the University of Cape Town, South Africa for processing and analysis.

### Recruitment

The cohort for this study was nested in a cervical cancer bioresource in Zimbabwe that was aimed at collecting data and enrolling donors that can be used in genomics studies [33]. Briefly, the cervical cancer bioresource recruited participants who were identified through the RTC registry. The inclusion criteria to participate in the cervical cancer bioresource were: (i) women aged > 18 years, (ii) histologically confirmed cervical cancer, (iii) accessible tumour tissue for analysis, and (iv) participants who were prescribed to receive radical cisplatin-based anticancer therapies. Individuals who had commenced anticancer therapy before recruitment into the bioresource were excluded, so as to enable data from the bioresource to be useful for future pharmacogenomics studies. Informed consent was administered verbally, and consent was provided in writing.

### Sampling

The sample size for this study was based on the availability of matched tumour and participant information in the cervical cancer bioresource [33] therefore, the power of the study was not computed. This article describes data from 240 study participants with histologically confirmed cervical cancer, who had complete matched clinical, sociodemographic and risk factor information and HPV genotype data.

### Data collection

Participant information on age, body mass index, marital status, history of STIs and HIV status were collected from the patient’s clinical records. Face-to-face interviews were used to collect data on behavioural risk factors such as alcohol consumption, tobacco smoking, menstrual cycle, age of first contraception, type of contraceptive, parity, circumcised sexual partner, age of sexual debut and self-reported history of herbal medication/insertions into the vaginal region were collected. All data were collected on a standardised paper-based tool.

### Outcome of cervical cancer treatment

The secondary objective of this study is to determine if distinct HR-HPVs are associated with the treatment outcomes, namely alive versus deceased. We collected data on the outcome of study participants from their patient records, at 12 months after they were prescribed to have completed anti-cancer treatment, as described in a previous study [34]. The outcome variable in this study was defined as the participant being alive or deceased 12-months after completing cervical cancer treatment.

### Tumour tissue retrieval and processing

Archived tumour tissue for all recruited participants was retrieved from the diagnosing pathology laboratories, in formalin-fixed paraffin embedded (FFPE) blocks. An equivalent of 4 microns of the FFPE blocks were sectioned off for analysis. Genomic DNA was extracted using the Zymo Genomic DNA for FFPE kit (Zymo Research, USA) as previously described [35]. Genomic DNA from the tumour was genotyped for 14 HR-HPVs using multiplex real-time PCR as we previously described [35]. This study detected for the presence of HR-HPVs: 16, 18, 31, 33, 35, 45, 51, 52, 56, 58, 59, 66 and 68. Only samples with an internal control (IC +) were included in these analyses.

### Statistical analysis

STATA v12.0 (StataCorp, Station College, TX, USA) and GraphPad Prism 8 (GraphPad Software Inc, California, USA) were used to analyse the data. Demographic and clinical data were allocated into either continuous and then expressed as mean ± standard deviation or median (interquartile range); or categorical data which was expressed as absolute or relative frequencies. Data on the outcome of treatment (alive versus deceased) was computed descriptively. The data was assessed for normality using the Shapiro–Wilk’s test. Differences in proportions for categorical data were analysed by computing Chi-squared test of homogeneity, using Pearson’s Chi-squared and Fisher’s exact tests as test statistics. Univariate and multivariate regression analyses were used to determine an association between the clinicopathological factors or outcomes and distinct HR-HPV genotypes. In univariate regression, distinct HR-HPV types were used as predictors in each regression model, and the clinicopathological factors or outcomes were covariates. In multivariate regression analyses, distinct HR-HPV genotypes were applied as predictors, and we built a single model with age, BMI, HIV status, history of smoking, history of STI, parity, age of menarche and the type of contraception to calculate adjusted odds ratios and 95% confidence intervals. Odds ratios and 95% confidence intervals were used as measures of association, and statistical significance was defined as *p* < 0.05. To determine factors associated with outcome (alive vs. deceased), a multivariable logistic regression model was built with the outcome as a predictor and covariates that included HPV genotype, age, BMI, HIV status, smoking history, and other factors that were found to be of significance univariable regression analysis. We computed odds ratios and 95% confidence intervals, as well as *p*-values.

## Results

The mean age (SD) was 52 (12) years (Table 1). More than half (51%) were within the normal BMI range (18.5–24.9 kg/m^2^) while 39.2% were overweight (25.0–29.9 kg/m^2^) or obese (> 30 kg/m^2^). Forty-eight percent of participants were HIV positive, and 30% had a positive history of STI. Squamous cell carcinoma (82%) was the most common histological type.

**Table 1:** Description of the sociodemographic and clinicopathology of the cohort

Characteristics	
Mean Age, in years (SD)	52 (12)
Mean Age of sexual debut, in years (SD)	19 (3)
Mean Age at first contraception, in years (SD)	20 (6)
Mean Age of menarche, in years(SD)	13 (6)
Mean Age of first conception mean (SD) (years)	16 (8)
Mean parity (SD)	5 (3)
	N (%)
BMI	
Underweight (<18.4)	23 (9.6)
Normal (X–Y 18.5–24.9)	123 (51.3)
Overweight (A–B 25–29.9)	83 (34.6)
Obese (>30)	11 (4.6)
Marital status	
Single (Never mind)	0 (0.0)
Married	100 (41.7)
Widowed	75 (31.3)
Divorced	65 (27.1)
Alcohol consumption, Yes	29 (12.1)
Tobacco Smoking, Yes	8 (3.3)
HIV positive, Yes	115 (48.0)
STI history	71 (29.6)
Number of sexual partners	
1	142 (60.0)
2–4	86 (35.0)
> 5	12 (5.0)
Circumcised partner	17 (7.0)
Types of Contraception	
Combined oral contraceptive	154 (64.0)
Implant	21 (9.0)
Injectable	36 (15.0)
Other	29 (12.0)
Herbal medicine use*	24 (10.0)
Tumour histology	
Squamous cell carcinoma	196 (81.7)
Adenocarcinoma	29 (12.1)
Adenosquamous carcinoma	8 (3.3)
Other**	7 (2.9)

*Self-reported history of herbal medicines inserted in the vaginal region. **other = basoloid, spindle cell, papillary serous and small cell cancers

### HR-HPV distribution

**Fig. 1:**
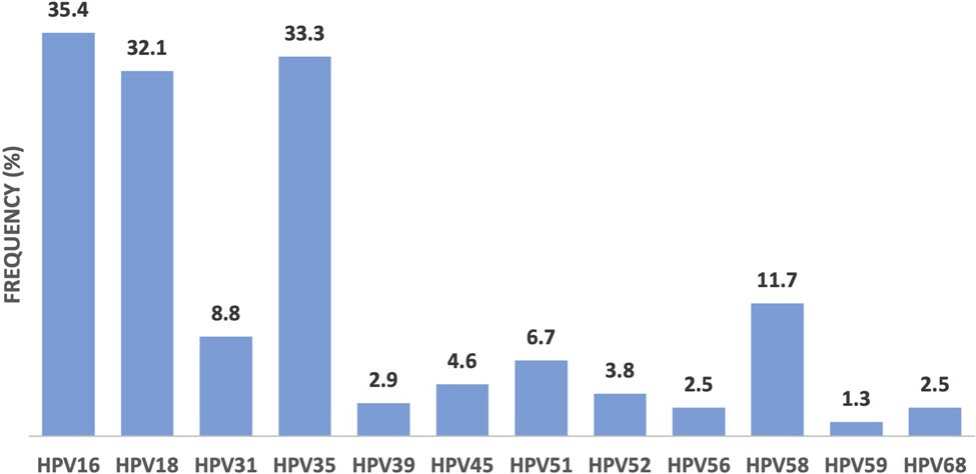
Frequency of HR-HPV genotypes

Distribution of the HR-HPV in this group of women with cervical cancer shows that HPV16 was the most common genotype (35%), followed by HPV35 (33%), HPV18 (32%) and HPV58 (12%) (Fig. 1). HPVs 33 and 66 were not detected in any of the tumour tissue analysed in this study.

In this study, 9 participants (3.8%) tested negative for any HR-HPVs assayed here despite the internal control being detected. A total of 92 (38.3%) presented with mono-infections, and the remainder (58.9%) presented with multiple HR-HPVs infections (Table 2).

**Table 2:** Number of HR-HPV genotype infections detected in this study

Number of HPVs	N (%)
0	9 (3.8)
1	92 (38.3)
2	76 (31.7)
3	45 (18.8)
4	13 (5.4)
5	2 (0.8)
6	3 (1.3)

### Association of clinicopathologic factors with HR-HPV genotypes in unadjusted analyses

**Fig. 2:**
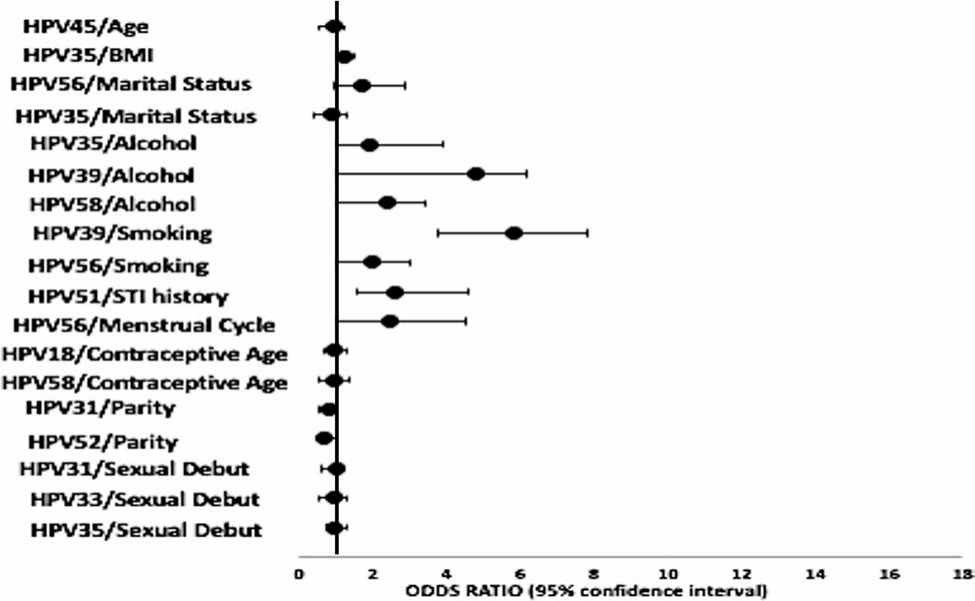
Factors associated with the distinct HPV genotypes. Odds Ratios and 95% confidence intervals (95% CI) for significant risk factors per specific HPV genotypes. Age* = age of contraceptive commencement

Unadjusted regression analysis was conducted to determine factors associated with positivity for specific HR-HPV genotypes (Supplementary Fig. 1) and only factors of statistical significance are illustrated in Fig. 2. Older age was associated with HPV45 (OR = 0.8; 95% CI 0.1–0.9, *p* = 0.032), while high BMI was associated with HPV35 (OR = 1.2; 95% CI 1.1–1.5, *p* = 0.040). Having a divorced/widowed marital status was associated with HPV56 (OR = 1.9; 95% CI 1.2–3.8, *p* < 0.001). A positive history of STIs was associated with HPV51 (OR = 2.5, 95% CI 1.8–4.4, *p* < 0.010). Alcohol consumption was associated with HPV35 (OR = 1.9, 95% CI 1.1–4.9, *p* = 0.049), HPV58 (OR = 2.5, 95% CI 1.6–3.8, *p* = 0.030) and HPV39 (OR = 5.8, 95% CI 2.0–7.8, *p* = 0.001). Smoking history was associated with HPV39 (OR = 6.4, 95% CI 4.1–8.3, *p* = 0.034) and HPV56 (OR = 2.0, 95% CI 1.2–4.3, *p* = 0.001).

Irregular menstrual cycle was associated with HPV56-positivity (OR = 2.5, 95% CI 1.3–8.5, *p* = 0.048). Starting contraceptives at an older age was associated with HPV18 (OR = 0.8; 95% CI 0.6–0.9, *p* = 0.025) and HPV58 (OR = 0.8; 95% CI 0.4–0.9, *p* = 0.042). Higher parity was associated with HPVs 31 (OR = 0.8; 95% CI 0.4–0.9, *p* = 0.035) and HPV52 (OR = 0.7; 95% CI 0.2–0.8, *p* = 0.024). Older age of sexual debut was is associated with reduced odds of positivity HPVs 31 (OR = 0.9; 95% CI 0.4–0.9, *p* = 0.044), HPV33 (OR = 0.9; 95% CI 0.4–1.0, *p* = 0.040), and HPV35 (OR = 0.9; 95% CI 0.7–0.9, *p* = 0.038).

### Association of HR-HPV genotypes with tumour histopathology

**Fig. 3:**
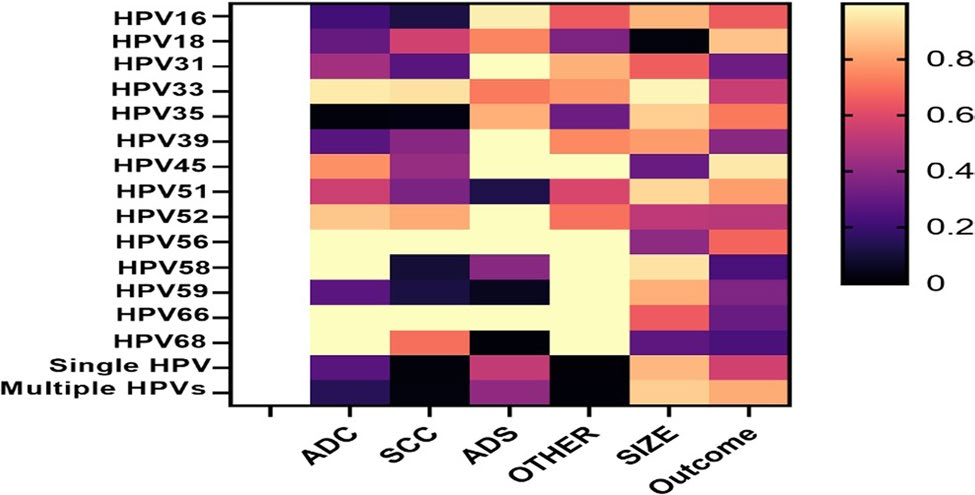
An association of tumour characteristics and treatment outcome with the distinct HR-HPV genotypes. Heatmap generated from *p*-values of univariate regression analyses of tumour characteristics and outcomes per HR-HPV genotypes. ADC = adenocarcinoma; SCC = squamous cell carcinoma; ADS = adenosquamous; Other = basoloid, spindle cell, papillary serous and small cell carcinomas

Univariate logistic regression analyses were used to determine the relationship between HR-HPVs with tumour characteristics, and the treatment outcome (Fig. 3).

### Association of HR-HPV genotypes with outcome

Of the 240 participants, 196 (81.7%) had treatment outcome data that was available for analysis. We found that 160 (81.6)% of participants were alive, and 36 (18.4%) were deceased 12 months after treatment completion. We conducted univariable logistic regression analysis to determine if there is an association between distinct HR-HPV genotypes and outcome i.e., whether the participant is alive or deceased 12 months after completing therapy. We found no association between HR-HPV genotypes and whether the study participant was alive or deceased (*p* > 0.05) (Fig. 3).

**Fig. 4:**
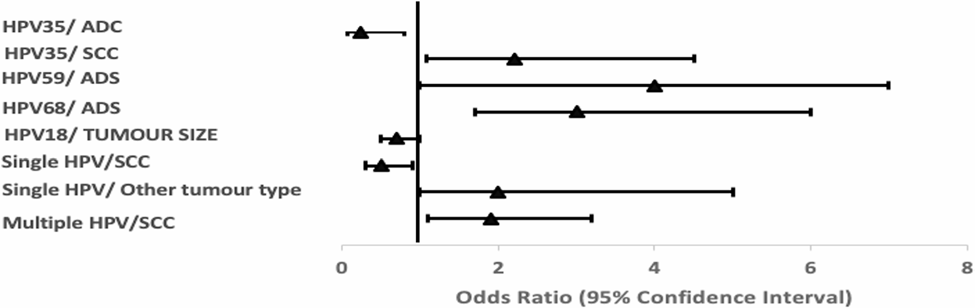
Forest plot illustrating odds ratio and 95% confidence interval for univariate regression analysis of HR-HPV genotypes with tumour characteristics. ADC: adenocarcinoma; SCC: squamous cell carcinoma; ADS: adenosquamous

HPV35 was associated with squamous cell carcinoma (OR = 2.2; 95% CI 1.4–7.0, *p* = 0.030) (Fig. 4). HPV59 (OR = 4.2; 95% CI 1.1–7.0, *p* = 0.020) and HPV68 (OR = 3.0; 95% CI 1.7–6.0, *p* = 0.010) were associated with adenosquamous carcinomas (Fig. 4). Single HPV infections were more likely observed in other histological tumour types (OR = 2.2; 95% CI 1.2–5.1, *p* = 0.010), while multiple HPV infections were more likely observed in squamous cell carcinoma (OR = 2.0; 95% CI 1.4–2.8, *p* < 0.010).

In adjusted analyses, high BMI (aOR:1.4; 95% CI 1.1–1.7, *p* = 0.010) was associated with HPV35 positivity (Table 3). Individuals with a history of smoking were more likely to have HPV68-positivity (aOR: 35.3; 95% CI 1.8–677.3, *p* = 0.018). There were no predictors for single versus multiple HPV infections (Table 3).

**Table 3:** Multivariate regression analysis illustrating factors associated with HR-HPVs

Predictor/covariates	aOR (95% CI)	*p*
HPV35		
Age	1.0 (1.0–1.0)	0.488
BMI	1.4 (1.1–1.7)	**0.010**
HIV	0.7 (0.4–1.4)	0.376
Smoking	3.7 (0.7–19.4)	0.070
ADC	0.7 (0.1–5.2)	0.951
SCC	3.7 (0.9–15.0)	0.057
ADS	4.2 (0.4–39.8)	0.118
STI history	0.8 (0.4–1.5)	0.417
Parity	0.9 (0.8–1.5)	0.276
Menarche	1.0 (0.9–1.0)	0.522
Type of contraception	1.0 (0.8–1.1)	0.526
HPV59		
Age	1.0 (0.9–1.1)	0.670
BMI	0.9 (0.4–2.6)	0.917
HIV	–	–
Smoking	–	–
STI history	4.2 (0.2–73.2)	0.324
Menarche	0.9 (0.7–1.1)	0.179
Parity	0.6 (0.2–1.5)	0.248
Type of contraceptive	0.8 (0.4–1.9)	0.625
HPV18		
Age	1.0 (1.0–1.0)	0.476
BMI	1.0 (0.8–1.3)	0.684
HIV	1.7 (0.9–3.0)	0.101
Smoking	1.6 (0.3–8.3)	0.545
STI history	1.0 (0.6–1.9)	0.955
Menarche	0.9 (0.9–1.0)	0.314
Parity	1.1 (1.0–1.3)	0.105
Type of contraceptive	1.0 (0.9–16.6)	0.801
HPV68		
Age	0.9 (0.9–1.0)	0.284
BMI	1.0 (0.4–2.1)	0.908
HIV	1.7 (0.2–16.0)	0.646
Smoking	35.3 (1.8–677.3)	**0.018**
STI history	0.9 (0.1–5.8)	0.918
Parity	0.8 (0.1–5.5)	0.822
Menarche	1.0 (0.8–1.1)	0.353
Type of contraceptive	1.0 (0.7–1.4)	0.915
Single vs multiple HPV infection		
Age	1.0 (1.0–1.1)	0.584
BMI	0.8 (0.4–1.4)	0.420
HIV	1.4 (0.3–6.5)	0.662
Smoking	–	–
STI history	0.4 (0.1–1.6)	0.205
Parity	0.9 (0.7–1.4)	0.764
Menarche	0.9 (0.7–1.1)	0.301
Type of contraceptive	1.1 (0.8–1.5)	0.627

## Discussion

This cross-sectional study describes factors associated with distinct HR-HPV genotype infections in a group of Zimbabwean women with cervical cancer. We found that BMI and smoking, were associated with distinct genital HR-HPVs, 35 and 68 respectively. This is the first known study to report an association between clinical and behavioural factors with HR-HPV positivity in Zimbabwean populations.

The relationship between BMI and HPV has been debated for a long time. Our study detected an association between high BMI and HPV35 positivity. Specific variants in the HPV35 type have been identified leading to distinctive drivers for persistence [36, 37]. It has been suggested that high BMI induces high inflammation in the genital tract, creating a conducive microenvironment for HPV persistence and carcinogenesis [38–40]. Although inflammation and tumour microenvironment were not considered in this study, there is need to analyse these relationships to understand possible pathways by which BMI may increase the odds of HPV35 positivity. Contrasting studies have found no association between HPV and BMI [41, 42]. However, these contraindicative data did not focus on specific HPV types, or in instances where HPVs were stratified, very low frequency of HPV genotypes that are not HPV16 and HPV18, thereby potentiating underpowered data.

In this study, tobacco smoking, was found to be associated with HPV35. Tobacco smoking induces transformation in the humoral immune response system by suppressing HPV antibody production, inhibiting T-cell activity and natural killer cells [43]. Over and above that, some studies show evidence of the presence of accumulated nicotine and other toxic composites from cigarette smoke in the cervical mucus of individuals with a history of smoking [44, 45]. Present findings support Kum-Nji and collaborators’ observations, of the significant association between tobacco smoking and susceptibility to HPVs such as HPV16, HPV18 and HPV35 [45]. Although some reports describe smoking to be a noticeable risk factor for HPVs 16 and 18 only, conflicting observations are pointing to a lack of differences in the risk of HPV acquisition and persistence between current smokers and previous smokers [46]. Our study did not distinguish between current smokers and previous smokers, even though this may be useful in exacting risk. Outcomes such as these can be applied to develop educational tools on risks of behavioural factors such as smoking in HPV persistence. Moreover, smoking has also been associated with an increased risk of squamous cell carcinoma, and not adenocarcinoma [47].

Parity is a hormone-regulating episode that has been previously associated with HPV. Data presented in this study did not find an association between parity and any of the HPVs. The median parity observed in the topical cohort was 4(3–6), which is comparable with the mean national parity (3.4) [48]. Similar findings have also described no association between parity and HR-HPV in previous studies, including a large multi-centre cohort study conducted by the International Agency on Research and Cancer [49, 50]. In contrast, other studies associate multiparity with HPV positivity and multiple HPVs infections [51, 52]. Various mechanisms are proposed to increase parity-induced susceptibility in relation to HPV because pregnancy has a high level of oestrogen [53]. Oestrogen induces cervical abnormalities including high HPV gene expression, suppressed immune response pathways, and accelerated cell proliferation in the transformation zone of the cervix [53]. In addition, the process of childbearing results in vaginal trauma, which also necessitates HPV infection and persistence [53]. Differences observed between our findings and other studies can be attributed to a low frequency in primiparity (n = 21) and nulliparity (n = 3) recorded in this cohort, potentiating the under-representation in comparative analysis. Therefore, a larger study with matched participants by parity can be utilized to confirm the role of parity in the risk of HPV and the distinctive genotypes.

In the present study, the distinct HPV genotypes were not associated with disease prognosis, resonating with previous publications [54, 55]. Contraindicative studies define HPVs 16, 18 and 31 as independent prognostic indicators for cervical cancer [30, 56]. Furthermore, poor prognosis is a complex sequela of clinical, stochastic, patient-centered factors, e.g., adherence, quality of life, socioeconomic status, and health-system challenges. Therefore, incongruous findings can be a result of compounding prognostic factors that differ between socioeconomic settings.

In addition, this study reported a lack of association between HPV DNA-negative tumours and prognosis. Similar findings have been reported in other data, leading to conclusions that HPV DNA status may be of minimal prognostic relevance [57]. Contrastingly, other data has found that HPV-negative tumours are more adversarial compared to HPV-positive tumours, resulting in more aggressive treatment regimens for successful curative outcomes [58, 59]. In this study, the lack of relationship may be a result of a low detection rate for the HPV-negative tumours (10 of 258), or the limited number of HPVs assayed in this study.

### Limitations

There are several limitations in interpreting our study results. Firstly, this study was an exploratory cross-sectional case-only study, thus, current findings may not be generalizable to exact risk of significance i.e., BMI and smoking for application in clinical practice. A prospective evaluation in a case–control design would be essential to validate the findings.

Secondly, our study did not consider the HPV vaccination status of the study participants. The national HPV vaccination program began in 2018 in Zimbabwe to target 10–14-year-olds [60] and given the mean age in this study (52 years ± 12), it is likely that most of the women probably had no prior history of HPV vaccination. Given the HPV vaccination data is becoming available, future studies should consider HPV vaccination status when analysing HPV genotype infections, and related factors associated.

Thirdly, our study did not compute survival analysis due to limitations in accessing records that indicate cause of death and date of death of participants. Further studies are needed to analyse survival analyses by HPV type towards understanding prognostic role of HPV.

## Conclusion

In conclusion, this data illustrate that biological, behavioural and gynecological characteristics are associated with type-specific HPV genotypes. Our findings underscore that implementing behavioural as well as therapeutic interventions in addition to the currently available vaccination programs are vital to reducing the HPV burden, and consequently, HPV-related cancers in countries such as Zimbabwe, where the prevalence is high. This study did not report an association between HPV genotypes and outcome of treatment, highlighting a need for further research with more robust survival analysis to determine if there is no prognostic significance of distinct HPV genotypes.

## Electronic supplementary material

Below is the link to the electronic supplementary material.

Supplementary Material 1

## Data Availability

No datasets were generated or analysed during the current study.

## References

[1] World Health Organisation. Cervical cancer. 2022. https://www.who.int/news-room/fact-sheets/detail/cervical-cancer Accessed on: 2 Oct 2023.

[2] Kutz JM, Rausche P, Gheit T, Puradiredja DI, Fuscp D. Barriers and facilitators of HPV vaccination in sub-Saharan Africa: a systematic review. BMC Public Health. 2923;23:974. 10.1186/s12889-023-15842-1 PMC10214362 37237329

[3] Isabirye A, Elwange BC, Singh K, Allegri MD. Individual and community-level determinants of cervical cancer screening in Zimbabwe: a multi-level analyses of a nationwide survey. BMC Womens Health. 2022;22:309. 35879710 10.1186/s12905-022-01881-0 PMC9310401

[4] Bruni L, Albero G, Serrano B, Mena M, Collado JJ, Gomez D, et al. ICO/IARC Information Centre on HPV and Cancer (HPV Information Centre). Human papillomavirus and Related Diseases in Zimbabwe. Summary Report 10 March 2023; https://hpvcentre.net/statistics/reports/ZWE.pdf.

[5] Kuguyo O, Matimba A, Tsikai N, Magwali T, Madziyire M, Gidiri M, et al. Cervical cancer in Zimbabwe: a situation analysis. Pan Afr Med J. 2017;27:215. 28979617 10.11604/pamj.2017.27.215.12994 PMC5622829

[6] Zibako P, Tsikai N, Manyame S, Ginindza TG. Cervical cancer management in Zimbabwe (2019–2020). PLoS ONE. 2022;17(9): e0274884. 36129898 10.1371/journal.pone.0274884 PMC9491541

[7] Bowden SJ, Doullgeraki T, Bouras E, Markozannes G, Athanasiou A, Grout-Smith H, et al. Risk factors for human papillomavirus infection, cervical intraepithelial neoplasia and cervical cancer: an umbrella review and follow-up Mendelian randomisation studies. BMC Med. 2023;21:274. 37501128 10.1186/s12916-023-02965-w PMC10375747

[8] Plesa A, Socolov D, Huica I, Botezatu A, Iancu IV, Ungureanu C, et al. High-risk human papillomaviruses distribution in Romanian women with negative cytology. J Infect Dev Ctries. 2019;13(4):326–33. 32045377 10.3855/jidc.11103

[9] Seyoum A, Assefa N, Gure T, Seyoum B, Mulu A, Mihret A. Prevalence and genotype distribution of high-risk human papillomavirus infection among sub-Saharan African women: a systematic review and meta-analysis. Front Public Health. 2022;10: 890880. 35875040 10.3389/fpubh.2022.890880 PMC9304908

[10] Rahangdale L, Mungo C, O’Connor S, Chibwesha CJ, Brewer NT. Human papillomavirus vaccination and cervical cancer risk. BMJ. 2022;379: e070115. 36521855 10.1136/bmj-2022-070115

[11] Zhao R, Sekar P, Bennis SL, Kulasingam S. A systematic review of the association between smoking exposure and HPV-related cervical cell abnormality among women living with HIV: implications for prevention strategies. Prev Med 2023; 170.107494. 10.1016/j.ypmed.2023.107494 37001607

[12] Hemmat N, Baghi HB (2019) Association of human papillomavirus infection and inflammation in cervical cancer. Pathogens Dis 2019;77(5):ftz048. 10.1093/femspd/ftz048 31504464

[13] Xi LF, Koutsky LA, Castle PE, Edelstein ZR, Meyers C, Ho J, et al. Relationship between cigarette smoking and human papillomavirus type 16 and 18 DNA load. Cancer Epidemiol Biomark Prev. 2010;18(12):3490–6. 10.1158/1055-9965.EPI-09-0763 PMC2920639 19959700

[14] Yang Z, Sub P, Dahlstrom KR, Gross N, Li G. Joint effect of human papillomavirus exposure, smoking and alcohol on risk of oral squamous cell carcinoma. BMC Cancer. 2023;23:457. 37202767 10.1186/s12885-023-10948-6 PMC10197209

[15] Laczano-Ponce E, Herrero R, Munoz N, et al. Epidemiology of HPV infection among Mexican women with normal cytology. Int J Cancer. 2001;91(3):412–20. 11169968 10.1002/1097-0215(20010201)91:3<412::aid-ijc1071>3.0.co;2-m

[16] Utami TW, Kusuma F, Winarto H, Anggraeni TD, Peters AAW, Spaans V, et al. Tobacco use and its association with HPV infection in normal uterine cervix: a study from a sustainable development goals perspective. Tob Induc Dis. 2021;19(64). 10.18332/tid/140093 PMC8340940 34413719

[17] Cooper D, Hoffman M, Carrara H, Rosenberg L, Kelly J, Stander I, et al. Determinants of sexual activity and its relation to cervical cancer risk among South African women. BMC Public Health. 2007;7:341. 18042284 10.1186/1471-2458-7-341 PMC2228293

[18] Ajuwon AJ, Olaleye A, Faromoju B, Ladipo O. Sexual behavior and experience of sexual coercion among secondary school students in three states in North-Eastern Nigeria. BMC Public Health. 2006;6:310. 17187685 10.1186/1471-2458-6-310 PMC1764888

[19] Manga MM, Fowotade A, Abdullahi YM, Ek-nafaty AU, Adamu DB, Pindiga HU, et al. Epidemiological patterns of cervical human papillomavirus infection among women presenting for cervical cancer screening in North-Eastern Nigeria. Infect Agent Cancer. 2015;10:39. 26435733 10.1186/s13027-015-0035-8 PMC4592568

[20] Thomas TL, Yarandi HN, Dalmida SG, Frados A, Klienert K. Cross-cultural differences and sexual risk behaviour of emerging adults. J Transcult Nurs. 2015;26(1):64–72. 24692340 10.1177/1043659614524791 PMC4182167

[21] Clarke MA, Risley C, Stewart MW, Geisinger KR, Hiser LM, Morgan JC, Owens KJ, Ayyalasomayajula K, Rive RM, Jannela A, et al. Age-specific prevalence of human papillomavirus and abnormal cytology at baseline in a diverse statewide prospective cohort of individuals undergoing cervical cance screening in Mississippi. Cancer Med. 2021;10(23):8641–50. 34734483 10.1002/cam4.4340 PMC8633239

[22] Mekonnen AG, Mittiku YM. Early onset of sexual activity as a potential risk of cervical cancer in Africa: a review of literature. PLOS Glob Public Health. 2023;3(3): e0000941. 36962975 10.1371/journal.pgph.0000941 PMC10032528

[23] Yin LX, D’Souza G, Westra WH, Wang SJ, va Zante A, Zhang Y, et al. Prognostic factors for human papillomavirus-positive and negative oropharyngeal carcinomas. The Laryngoscope. 2018;128(8):288–296. 10.1002/lary.27130 PMC8929688 29536542

[24] Huang Y, He Q, Xu K, Zhou J, Yin J, Li F, et al. A new marker based on risk stratification of human papillomavirus DNA and tumour size to predict survival of locally advanced cervical cancer. Int J Gynecol Can. 2019;29:459–65. 10.1136/ijgc-2018-000095 30733276

[25] Lin G, Yang LY, Lin YC, et al. Prognostic model based on magnetic resonance imaging, whole-tumour apparent diffusion coefficient values and HPV genotyping for stage IB-IV cervical cancer patients following chemoradiotherapy. Eur Radiol. 2019;29:556–65. 30051142 10.1007/s00330-018-5651-4

[26] Rose BR, Thompson CH, Simpson JM, Jarrett CS, Elliott PM, Tattersall MH, et al. Human papillomavirus deoxyribonucleic acid as a prognostic indicator in early-stage cervical cancer: a possible role for type 18. Am J Obstet Gynecol. 1995;173:1461–8. 7503185 10.1016/0002-9378(95)90633-9

[27] Nakagawa S, Yoshikawa H, Onda T, Kawana T, Iwamoto A, Taketani Y. Type of human papillomavirus is related to clinical features of cervical carcinoma. Cancer (Phila).1998;78:1935–41. 8909314

[28] Schwartz M, Daling JR, Shera KA, Madeleine MM, McKnight B, Galloway DA, et al. Human papillomavirus and prognosis of invasive cervical cancer: a population-based study. J Clin Oncol. 2001;19(7):1906–15. 11283122 10.1200/JCO.2001.19.7.1906

[29] Im SS, Wilczynski SP, Burger RA, Monk BJ. Early stage cervical cancers containing human papillomavirus type 18 DNA have more nodal metastasis and deeper stromal invasion. Clin Cancer Res. 2003;9:4145–50. 14519638

[30] Huang LW, Chao SL, Hwang JL. Human papillomavirus-31-related types predict better survival in cervical carcinoma. Cancer. 2004;100(2):327–34. 14716768 10.1002/cncr.20003

[31] Van Bommel PF, van den Brule AJ, Helmerhorst TJ, Gallee MP, Gaarenstroom KN, Walboomers JM, et al. HPV DNA presence and HPV genotypes as prognostic factors in low-stage squamous cell cervical cancer. Gynecol Oncol. 1993;48:333–7. 8385059 10.1006/gyno.1993.1058

[32] Wright JD, Li J, Gerhard DS, Zhang Z, Huettner PC, Powell MA, et al. Human papillomavirus type and tobacco use as predictors of survival in early-stage cervical carcinoma. Gynecol Oncol. 2005;98(1):84–91. 15894364 10.1016/j.ygyno.2005.03.038

[33] Kuguyo O, Chambwe N, Nhachi CFB, Tsikai N, Dandara C, Matimba A. A cervical cancer biorepository for pharmacogenomics research in Zimbabwe. BMC Cancer. 2022;22:1320. 36526993 10.1186/s12885-022-10413-w PMC9756582

[34] Kuguyo O, Matimba A, Madziyire MG, et al. Prevalence and predictors for cisplatin-induced toxicities in Zimbabwean women with cervical cancer. Future Oncol. 2024;20:1909–24. 39056302 10.1080/14796694.2024.2375959 PMC11498005

[35] Kuguyo O, Dube Mandishora RS, Thomford NE, Makunike Mutasa R, Nhachi CFB, Matimba A, et al. High-risk HPV genotypes in Zimbabwean women with cervical cancer: comparative analyses between HIV-negative and HIV positive women. PLoS ONE. 2021;16(9): e035732. 10.1371/journal.pone.0257324 PMC8478215 34582476

[36] Gagnon S, Hankins C, Tremblay C, Forest P, Pourreaux K, Coutlee F and Canadian Women’s HIV study group. Viral polymorphism in human papillomavirus types 33, 35 and persistent and transient infection in the genital tract of women. J Infect Dis. 2004;190(9):1575–1585. 10.1086/424854 15478061

[37] Mboumba Bouassa RS, Ntsigouaye JA, Tsimba PCL, Nodjikouambaye ZA, et al. Genetic diversity of HPV35 in Chad and the Central African Republic, two landlocked countries of Central Africa: A cross sectional study. PLoS ONE. 2024;19(1):e0297054. 10.1371/journal.pone.0297054 PMC10810494 38271382

[38] Hammes LS, Tekmal RR, Naud P, et al. Macrophages inflammation and risk of cervical intraepithelial neoplasia (CIN) progression—clinicopathological correlation. Gynecol Onol. 2007;105:157–65. 10.1016/j.ygyno.2006.11.023 17229459

[39] Fernandes JV, De Medeiros Fernandes TA, De Azevedo JC, Cobucci RN, De Carvalho MG, Andrade VS, De Arauj JM. Link between chronic inflammation and human papillomavirus-induced carcinogenesis (Review). Oncol Lett. 2015;9(3):1015–26. 25663851 10.3892/ol.2015.2884 PMC4315066

[40] Mastrogeorgiou M, Chatzkalil E, Theocharis S, Papoudou A, Peoc’h M, Mobarki M, Karpathiou G. The immune microenvironment of cancer of the uterine cervix. Histol Histopathol. 2024;39(10):1245–1271. 10.14670/HH-18-727 38483012

[41] Wee CW, Huang A, Huskey KW, McCarthy EP. Obesity and the likelihood of sexual behaviour risk factors for HPV and cervical cancer. Obesity (Silver Spring). 2008;16(11):2552–5. 18719677 10.1038/oby.2008.394 PMC2801345

[42] Urbute A, Thomsen LT, Belmonte F, Kesmodel US, Frederiksen K, Kjoer SK. The role of body mass index in incidence and persistence of cervical human papillomavirus infection. Ann Epidemiol. 2020;49:36–41. 32711054 10.1016/j.annepidem.2020.07.011

[43] Aguayo F, Munoz JP, Perez-Dominguez F, Carrillo-Beltran D, Oliva C, Calaf GM, et al. High-risk human papillomavirus and tobacco smoke interactions in epithelial carcinogenesis. Cancers (Basel). 2020;12(8):2201. 32781676 10.3390/cancers12082201 PMC7465661

[44] Prokopczyk B, Cox JE, Hoffmann D, Waggoner SE. Identification of tobacco-specific carcinogen in the cervical smokers and nonsmokers. J Natl Cancer Inst. 1997;89(12):868–73. 9196253 10.1093/jnci/89.12.868

[45] Kum-Nji P, Meloy L, Keyser-Marcus L. Tobacco smoke exposure as a risk factor for human papillomavirus infections in women 18–26 years old in the United States. PLoS ONE. 2019;14(10): e0223532. 31665134 10.1371/journal.pone.0223532 PMC6821098

[46] Utami TW, Kusuma F, Winarto H, et al. Tobacco use and its association with HPV infection in normal uterine cervix: a study from a sustainable development goals perspective. Tob Induc Dis. 2021;19(64). 10.18332/tid/140093 PMC8340940 34413719

[47] Berrington de Gonzalez A, Sweetland S, Green J. Comparison of risk factors for squamous cell and adenocarcinomas of the cervix: a meta-analysis. Br J Cancer. 2004;90(9):1787–1791. 10.1038/sj.bjc.6601764 PMC2409738 15150591

[48] Bruni L, Albero G, Serrano B, Mena M, Collado JJ, Gomez D, et al. ICO/IARC Information Centre on HPV and Cancer (HPV Information Centre). Human papillomavirus and related diseases report: World. 2022. https://hpvcentre.net/statistics/reports/XWX.pdf Accessed on: 21 May 2022.

[49] Munoz N, Franceshi S, Bosetti C, et al. Role of parity and human papillomavirus in cervical cancer: the IARC multicentric case-control study. Lancet. 2002;359:1093–101. 11943256 10.1016/S0140-6736(02)08151-5

[50] Castellsague X, Munoz N. Chapter 3: Cofactors in human papillomavirus carcinogenesis—role of parity, oral contraceptives, and tobacco smoking. J Natl Cancer.2003;31:20–8. 12807941

[51] Matos A, Moutinho J, Pinto D, et al. The influence of smoking and other cofactors on the time to onset to cervical cancer in a southern European population. Eur J Cancer Prev. 2005;14(5):485–91. 16175054 10.1097/01.cej.0000174780.44260.32

[52] Luhn P, Walker J, Schiffman M, et al. The role of co-factors in the progression from human papillomavirus infection to cervical cancer. Gynecol Oncol. 2013;128(2):265–70. 23146688 10.1016/j.ygyno.2012.11.003 PMC4627848

[53] Tekalegn Y, Sahiledengle B, Woldeyohannes D, et al. High parity is associated with increased risk of cervical cancer: systematic review and meta-analysis of case-control studies. Women’s Health. 2022;18. 10.1177/17455065221075904 PMC8819811 35114865

[54] Tong SY, Lee YS, Park JS, Namkoong SE. Human papillomavirus genotype as a prognostic factor in carcinoma of the uterine cervix. IJGC. 2007;17(6):1307–13. 17425678 10.1111/j.1525-1438.2007.00933.x

[55] Genta ND, Martins TR, Lopez RVM, et al. Multiple HPV genotype infection impact on invasive cervical cancer presentation and survival. PLoS ONE. 2017;12(8): e0182854. 28829791 10.1371/journal.pone.0182854 PMC5567480

[56] Lombard I, Vincent-Salomon A, Validire P, Zafrani B, de la Rochefordiere A, Clough K, et al. Human papillomavirus genotype as a major determinant of the course of cervical cancer. J Clin Oncol. 1998;16(8):2613–9. 9704710 10.1200/JCO.1998.16.8.2613

[57] Fernandes A, Viveros-Carreno D, Hoegi J, Avila M. Pareja R (2022) Human papilomavírus-independent cervical câncer. Int J Gynecol Cancer. 2022;32:1–7. 34725203 10.1136/ijgc-2021-003014

[58] Rodriguez-Carunchio L, Soveral I, et al. HPV-negative carcinoma of the uterine cevric: a distinctive of cervical cancer with poor prognosis. BJOG. 2015;122:199–227. 25229645 10.1111/1471-0528.13071

[59] Li P, Tan Y, Zhu LX, et al. Prognostic value of HPV DNA status in cervical cancer before treatment: a systematic review and meta-analysis. Oncotarget. 2017;8(39):66352–9. 29029517 10.18632/oncotarget.18558 PMC5630417

[60] LaMontagne DS, Manangazira P, Marembo J, et al. HPV vaccination coverage in three districts in Zimbabwe following national introduction of 0,12 month schedule among 10 to 14 year old girls. Vaccine. 2022;40(1):A58-66. 34275674 10.1016/j.vaccine.2021.07.012

