# Supplementary material for: Association between human papillomavirus and behaviour, clinicopathology, and cervical cancer outcome in Zimbabwean women: a cross-sectional study
Source: Reprod Health. 2025 Oct 7;22:190. doi: 10.1186/s12978-025-02100-3 (PMC12505816; doi:10.1186/s12978-025-02100-3)
Supplement: Supplementary file 1 — Supplementary Material 1 [file 12978_2025_2100_MOESM1_ESM.docx]

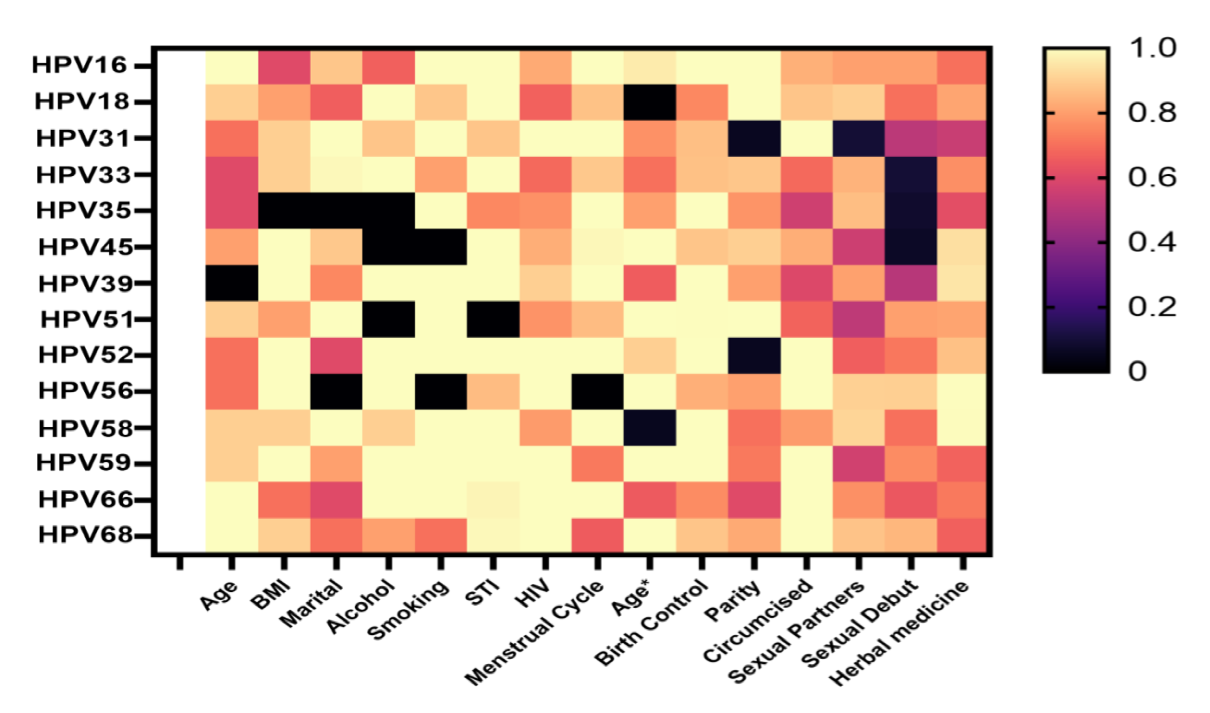


**Supplementary Figure 1:** **Factors associated with the distinct HPV genotypes**. Heatmap of associated risk factors of HPVs based on p-values calculated from regression analyses. Age*= age of contraceptive commencement.
